# Slow Wave Sleep Is a Promising Intervention Target for Alzheimer’s Disease

**DOI:** 10.3389/fnins.2020.00705

**Published:** 2020-06-30

**Authors:** Yee Fun Lee, Dmitry Gerashchenko, Igor Timofeev, Brian J. Bacskai, Ksenia V. Kastanenka

**Affiliations:** ^1^Department of Neurology, MassGeneral Institute of Neurodegenerative Diseases, Massachusetts General Hospital and Harvard Medical School, Charlestown, MA, United States; ^2^Department of Anatomy and Neurobiology, Boston University School of Medicine, Boston, MA, United States; ^3^Harvard Medical School/VA Boston Healthcare System, West Roxbury, MA, United States; ^4^Department of Psychiatry and Neuroscience, School of Medicine, Université Laval, Québec, QC, Canada; ^5^CERVO Brain Research Center, Québec, QC, Canada

**Keywords:** Alzheimer’s disease, sleep, NREM sleep, slow wave activity, slow oscillations

## Abstract

Alzheimer’s disease (AD) is the major cause of dementia, characterized by the presence of amyloid-beta plaques and neurofibrillary tau tangles. Plaques and tangles are associated with sleep-wake cycle disruptions, including the disruptions in non-rapid eye movement (NREM) slow wave sleep (SWS). Alzheimer’s patients spend less time in NREM sleep and exhibit decreased slow wave activity (SWA). Consistent with the critical role of SWS in memory consolidation, reduced SWA is associated with impaired memory consolidation in AD patients. The aberrant SWA can be modeled in transgenic mouse models of amyloidosis and tauopathy. Animal models exhibited slow wave impairments early in the disease progression, prior to the deposition of amyloid-beta plaques, however, in the presence of abundant oligomeric amyloid-beta. Optogenetic rescue of SWA successfully halted the amyloid accumulation and restored intraneuronal calcium levels in mice. On the other hand, optogenetic acceleration of slow wave frequency exacerbated amyloid deposition and disrupted neuronal calcium homeostasis. In this review, we summarize the evidence and the mechanisms underlying the existence of a positive feedback loop between amyloid/tau pathology and SWA disruptions that lead to further accumulations of amyloid and tau in AD. Moreover, since SWA disruptions occur prior to the plaque deposition, SWA disruptions may provide an early biomarker for AD. Finally, we propose that therapeutic targeting of SWA in AD might lead to an effective treatment for Alzheimer’s patients.

## Introduction

Alzheimer’s disease (AD) is a progressive neurodegenerative disorder and the most common cause of dementia in the elderly (Alzheimer’s Association, 2016). The pathological hallmarks of AD are the presence of extracellular plaques composed of amyloid-beta (Aβ) and intracellular neurofibrillary tangles composed of the microtubule binding protein tau in the brain (Bloom, 2014; Calderon-Garcidueñas and Duyckaerts, 2017). According to the amyloid cascade hypothesis, Aβ accumulation leads to tau deposition, triggers neuronal dysfunction and results in neuronal death (Hardy and Higgins, 1992; Selkoe and Hardy, 2016). Although, amyloid cascade hypothesis is widely debated, soluble Aβ and tau protein aggregations have been shown to lead to synaptic dysfunction and loss of synaptic density (Spires-Jones and Hyman, 2014), resulting in memory and cognitive deficits in AD patients (DeKosky and Scheff, 1990). The clinical features of AD include progressive memory loss, impaired judgment and decision-making (Förstl and Kurz, 1999). Current therapeutics are limited to alleviation of symptoms, not reversing or slowing the disease progression (Yiannopoulou and Papageorgiou, 2013; Cummings et al., 2019). Therefore, there is an urgent need to identify effective treatment strategies for alleviating the disease burden.

In addition to memory and cognitive impairments, Alzheimer’s patients experience sleep disruptions (Bliwise et al., 1995; McCurry et al., 1999; Moran et al., 2005), leading to reductions of non-rapid eye movement (NREM) sleep and slow wave activity (SWA), a brain rhythm prevalent during NREM sleep (Prinz et al., 1982). These disruptions include increased amounts and frequencies of nighttime wakefulness as well as daytime napping (Vitiello and Prinz, 1989; McCurry et al., 1999). Sleep disturbances manifest early since individuals with mild cognitive impairment (MCI), a pre-clinical stage of AD, suffer from sleep disruptions (Vitiello and Prinz, 1989; Westerberg et al., 2012). Similarly, individuals with detectable amyloid beta, but cognitively healthy also suffer from sleep disturbances, defined as lower sleep quality and increased number of day-time naps (Ju et al., 2013). Thus, sleep and memory disruptions manifest early in the disease progression, prior to symptom onset.

Sleep mediates several forms of memory consolidation (Diekelmann and Born, 2010; Born and Wilhelm, 2012; Rasch and Born, 2013). Sleep disturbances are correlated with deteriorated memory function and cognitive decline in AD and MCI patients (Moe et al., 1995; Brzecka et al., 2018). Increased night-time wakefulness and decreased slow wave sleep (SWS), which is dominated by SWA, was associated with impaired memory and cognitive functions (Moe et al., 1995). NREM, in particular SWS, plays an important role in declarative memory consolidation (Walker, 2009; Lu and Göder, 2012). This review will provide an overview of the SWA disruptions in AD. It will also summarize the evidence for the causal relationship between AD pathology, Aβ/tau, and sleep-dependent memory consolidation deficits that are driven by the SWA disturbances in AD patients and animal models of AD. Furthermore, we will propose possible mechanisms underlying the SWA disruptions. Finally, we will discuss therapeutic strategies for targeting SWS in AD aimed at slowing the disease progression and restoring the sleep-dependent memory consolidation. This review is focused on the SWA, the most prominent neocortical activity with the increased power density in 0.5–4.0 Hz frequency range occurring during NREM sleep. It will not cover other NREM sleep-associated rhythms, such as thalamo-cortical sleep spindles, or hippocampal ripples, nor will it discuss REM sleep disruptions in AD, that have been reviewed or described elsewhere (Christos, 1993; Rauchs et al., 2008; Pase et al., 2017).

## SWA and Sleep-Dependent Memory Consolidation

Sleep consists of rapid eye movement (REM) and NREM sleep. REM sleep is characterized by desynchronized EEG activity with faster oscillations and lower voltage waveforms (Carskadon and Dement, 2011). Human NREM sleep is subdivided into stages N1–N3 (previously stages 1–4) and is defined by the electroencephalogram (EEG) activity as synchronous waveforms, including sleep spindles (12–14 Hz), K-complexes in stage 2 as well as slow (<1 Hz) and delta (1–4 Hz) activity in stage 3 (Iber et al., 2007). Slow and delta oscillations or isolated slow waves are commonly called SWA (Timofeev et al., 2020). Stage N3, also referred to as Delta Sleep or SWS, is characterized by the high amounts of SWA (Carskadon and Dement, 2011).

Slow oscillation is a major rhythm of deep sleep. During slow oscillations, excitatory and inhibitory neocortical neurons from all layers (unknown for layer 1) in anesthetized (Steriade et al., 1993a,b,c) and sleeping animals (Timofeev et al., 2000b, 2001; Steriade et al., 2001; Chauvette et al., 2010) oscillate between depolarized (active or UP) and hyperpolarized (silent or DOWN) states. Despite involvement of the entire thalamocortical system (Steriade et al., 1993a; Contreras and Steriade, 1995; Sheroziya and Timofeev, 2014), the slow oscillations originate in neocortex as can be recorded in neocortical slices (Sanchez-Vives and McCormick, 2000; Sanchez-Vives et al., 2010), cortical cell cultures (Sun et al., 2010; Hinard et al., 2012) and isolated cortical slabs maintained *in vivo* (Timofeev et al., 2000a; Lemieux et al., 2014). Slow oscillation is absent from the thalamus of decorticated animals (Timofeev and Steriade, 1996). The silent (hyperpolarized or DOWN) states of slow oscillations are periods of disfacilitation, i.e., absence of synaptic activity. Leak currents primary mediate silent states (Timofeev et al., 1996, 2001). The active (depolarized or up) states are mediated by barrages of excitatory and inhibitory synaptic activities at the level of soma (Steriade et al., 2001; Timofeev et al., 2001; Rudolph et al., 2007) and major Ca^2+^ activities in dendrites (Milojkovic et al., 2007; Seibt et al., 2017). Neocortex generates slow oscillations while thalamus contributes to their maintenance as thalamic inactivation temporally modifies cortical SWA (David et al., 2013; Lemieux et al., 2014).

To control the slow oscillations, it is important to understand the major cellular events taking place during SWA. The neuronal firing and thus synaptic activity in local cortical networks, is essentially absent in the silent state. Two major mechanisms for the active state onset are proposed. (i) The silent state is partially mediated by Ca^2+^- and Na^+^-dependent K^+^ currents. A reduction in these currents leads to the onset of a new active state (Sanchez-Vives and McCormick, 2000). (ii) Silent states are characterized by the absence of synaptic activity, but spike-independent neurotransmitter release (miniature postsynaptic potentials, minis) are still present. Co-occurrence of minis in large neurons that possess a high number of postsynaptic sites can lead to significant depolarizations and initiations of spikes, that would drive the whole network into an active state (Timofeev et al., 2000a; Bazhenov et al., 2002; Chauvette et al., 2010). Since this is a stochastic process, it can start in any cell, but more often, it starts in larger neurons, typically layer 5 large cortical pyramidal cells in experimental animals (Chauvette et al., 2010; Fiáth et al., 2016). In human, however, slow wave active states more often start in layer 3 (Cash et al., 2009; Csercsa et al., 2010). There might be two reasons for this difference: (a) human pyramidal cells from layer 3 are very large (Mohan et al., 2015), and therefore, they are well situated to summate minis and to trigger active states; and (b) enhanced electrical compartmentalization in layer 5 pyramidal neurons in humans does not allow dendritic depolarizing events to reach soma, even in the presence of dendritic spikes (Beaulieu-Laroche et al., 2018), therefore reducing overall implication of layer 5 cells in network operation. Local origin of active states and dense synaptic interactions in the cortex trigger propagation of slow waves across cortical mantle (Massimini et al., 2004; Volgushev et al., 2006; Sheroziya and Timofeev, 2014). Active states are mediated by interactions of excitatory and inhibitory conductances (Haider et al., 2006; Haider and McCormick, 2009; Chen et al., 2012) with overall stronger inhibition at the level of soma (Rudolph et al., 2007; Haider et al., 2013). A termination of active states and transition to silent states occurs due to several factors: (i) activation of Na^+^- and Ca^2+^-dependent potassium currents (Sanchez-Vives and McCormick, 2000), (ii) synaptic depression (Timofeev et al., 2000b), and (iii) synchronous active inhibitory drive (Steriade et al., 1993b; Lemieux et al., 2015). Because active states terminate nearly simultaneously across large cortical territories (Volgushev et al., 2006; Sheroziya and Timofeev, 2014), intrinsic current activation or synaptic depression likely do not play a leading role, because they are cell specific. Thus, we suggest that active inhibitory mechanisms terminate active states and provide network-wide synchronous onset of silent states. First, somatostatin-positive GABAergic interneurons increase activity prior to the onset of silent states (Funk et al., 2017; Niethard et al., 2018). Most of these interneurons have short axons, therefore an external trigger, possibly from thalamus, synchronizes them. Indeed, thalamic inactivation abolishes synchronous onset of silent states (Lemieux et al., 2015). Furthermore, some thalamocortical neurons fire during silent states driving parvalbumin-positive interneurons (Zucca et al., 2019). Another potential source is claustrum, the structure that has widespread cortical projections and, if activated optogenetically, induces cortical down states (Narikiyo et al., 2018). It appears that claustrum is well situated to drive simultaneously cortical interneurons across different areas just prior to the onset of silent states.

Sleep slow oscillations play an important role in cortical plasticity. However, the direction of these plastic changes is still under discussion. A subset of studies, based mainly on indirect measurements, propose that cortical synaptic connections are strengthened during wakefulness and are weakened during sleep (Tononi and Cirelli, 2003, 2014). Other studies demonstrate that slow oscillations and overall sleep strengthens efficacy of cortical synapses (Aton et al., 2009; Chauvette et al., 2012; Seibt et al., 2012; Yang et al., 2014; Jasinska et al., 2015; Timofeev and Chauvette, 2017). Finally, there is an attempt to reach agreement in this debate which proposes that some synapses are upregulated and others are downregulated by sleep (Seibt and Frank, 2019).

Irrespective of the synaptic mechanisms, slow oscillations play an important role in sleep-dependent declarative memory consolidation (Steriade and Timofeev, 2003; Marshall et al., 2006; Walker, 2009; Lu and Göder, 2012). Born and colleagues proposed a model of declarative, hippocampus-related memory consolidation during SWS. Cortical slow oscillations drive the reactivation of short-term hippocampal memories by synchronizing hippocampal sharp wave ripples with spindle activity in the thalamus during slow oscillation UP states. This mechanism thereby contributes to the long-term synaptic plasticity changes in neocortical networks and supports the consolidation of long-term memory in neocortex (Diekelmann and Born, 2010; Rasch and Born, 2013). SWS declines with increasing age especially after the age of 30 (Van Cauter et al., 2000). Age-related reduction in SWS was correlated with impaired sleep-associated memory consolidation (Backhaus et al., 2007). Furthermore, insomnia patients with less SWS showed declines in overnight declarative memory consolidation compared to age-matched controls (Backhaus et al., 2006). Boosting SWA facilitated sleep-dependent memory consolidation (Marshall et al., 2006). Thus, SWA, slow oscillations in particular, is necessary and sufficient for memory consolidation during sleep, and we propose that SWA disruptions might contribute to memory impairments in AD.

## SWA Disruptions in AD Patients

**FIGURE 1:**
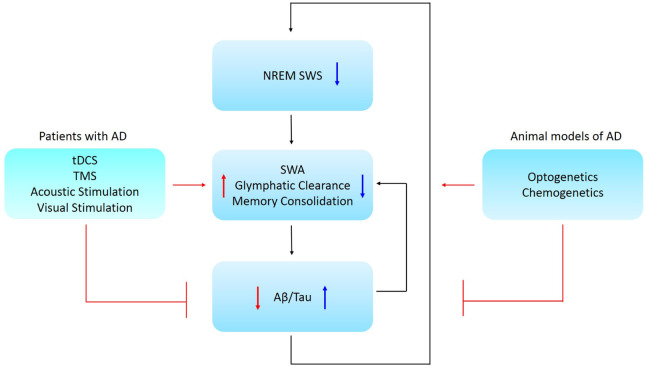
Schematic representation of the causal relationship between NREM SWS, memory consolidation, and AD pathology. Low NREM SWS leads to reduced SWA and perivascular clearance of soluble Aβ and is associated with impaired memory consolidation. Further, low SWA is associated with increased Aβ/tau aggregation in AD patients. Ways to restore NREM SWA are proposed for animal models and Alzheimer’s patients, where these might lead to a promising therapeutic strategy for AD. Abbreviations: NREM SWS, non-rapid eye movement slow wave sleep; SWA, slow wave activity; Aβ, amyloid-beta; AD, Alzheimer’s disease; tDCS, transcranial direct current stimulation; TMS, transcranial magnetic stimulation.

Toxic Aβ is thought to initiate pathological events and drive the formation of pathological tau aggregates that ultimately lead to synaptic loss and cell death (Hardy and Higgins, 1992; Selkoe and Hardy, 2016; Henstridge et al., 2019), which in turn compromises neuronal circuitry. Aβ levels correlate with sleep alterations in cognitively normal individuals with preclinical AD (Ju et al., 2013; Spira et al., 2013). Neuronal activity disturbances including slow oscillation disruptions were reported in older adults (Mander et al., 2013, 2015; Lucey et al., 2019). Aβ and tau deposits were associated with decreased NREM SWA in cognitively normal older adults and in early stages of AD (Mander et al., 2015; Lucey et al., 2019). Decreased NREM slow oscillations (0.6–1 Hz) were associated with increased Aβ accumulation in the medial prefrontal cortex (Mander et al., 2015). Also, higher tau deposition was correlated with decreased delta power (1–4 Hz) (Lucey et al., 2019). This evidence provides strong support for a relationship between SWA disruptions and AD pathology (Figure 1). In addition to SWA disruptions in asymptomatic cognitively normal adults, SWA was reduced in MCI individuals (Westerberg et al., 2012). Sleep disturbances in cognitively normal older adults could predict the Aβ burden and tau accumulation later in life (Winer et al., 2019). Taking into account all of the above, we propose that, sleep-wake cycle disturbances, especially decreases in NREM SWA, may serve as a potential early biomarker for AD.

Aberrations in SWA correlated with disrupted memory consolidation in MCI patients (Westerberg et al., 2012) and older adults (Mander et al., 2015). Therefore, it stands to reason that slow oscillation disruptions in individuals with early AD might contribute to and accelerate the progressive memory and cognitive decline. In turn, AD pathology might further disrupt sleep-dependent brain rhythm activity that further exacerbates AD (Figure 1).

Glymphatic system hypothesis, although still debated (Abbott et al., 2018), states that soluble Aβ is cleared along perivascular pathways, including through the glymphatic system (Iliff et al., 2012). SWS enhanced the clearance of Aβ when compared to the waking state (Xie et al., 2013). Interestingly, soluble Aβ levels fluctuated with the sleep-wake cycle in humans. Aβ levels were elevated during waking and declined during sleep (Kang et al., 2009; Lucey et al., 2017), suggesting that sleep facilitates Aβ clearance. Thus, there is accumulating evidence for a relationship between Aβ, sleep and neuronal activity disruptions (Figure 1).

## SWA Disruptions in Mouse Models of AD

Mouse models of AD provide a powerful means to study SWA disruptions. Amyloidosis models recapitulate Aβ production, amyloid plaque deposition and associated neuropathology exhibited by Alzheimer’s patients. Tauopathy models mimic tau production, intracellular tau tangles and associated neuropathology. Furthermore, slow oscillation disruptions were recapitulated in mouse models of amyloidosis and tauopathy (Holth et al., 2017; Kastanenka et al., 2017; Castano-Prat et al., 2019). Wide field imaging using voltage sensitive dyes (VSD) and genetically encoded reporters, in addition to traditional methods, such as electrophysiology, allowed monitoring SWA in mice. Similar to those in humans (Massimini et al., 2004), slow oscillations propagate across cortex in mice as traveling waves between two hemispheres (Mohajerani et al., 2010). We used a transgenic mouse model of amyloidosis (APPswe/PS1dE9 mice; APP mice) to show that the cortical slow wave power but not the frequency was decreased in young (Kastanenka et al., 2017) and older mice (Kastanenka et al., 2019). APP mice spent less time in NREM sleep (Roh et al., 2012). Tg2576 mice exhibited decreases in SWA; and their power spectral density was shifted to higher frequencies (Kent et al., 2018). As for tauopathy models, P301S human tau transgenic mice exhibited sleep-wake cycle disruptions, reductions in NREM sleep and increased wakefulness. Moreover, their SWA was significantly decreased during NREM sleep (Holth et al., 2017). Furthermore, the transgenic mouse model 3xTg-AD, which develops plaque and tangle pathology, exhibited slow waves at lower frequency and reduced firing rate (Castano-Prat et al., 2019). Thus, these animal models recapitulated SWA disruptions exhibited by Alzheimer’s patients (Lucey et al., 2019). It should be noted that animal models do not recapitulate all aspects of human condition. Mice have more primitive cortex and hence slow oscillations present in mice are not as complex as those recorded in humans. During aging, sleep in mice undergoes changes, sometimes dissimilar to those in humans. For example, aged mice exhibit less SWS compared to young mice. The power of slow-wave activity in aging mice is increased when measured in frontal cortex, while slow wave power in aging human adults is decreased. On the other hand, aging mice also exhibit similarities to aging humans. For example, aged mice exhibit increases in sleep fragmentation, increases in sleep duration during active phase of sleep-wake cycle (light for humans, dark for mice), and decreased REM sleep at the end of quiet phase of sleep-wake cycle (Soltani et al., 2019). Thus use of mouse models should be considered with caution when modeling human condition. Despite these limitations, the mouse models were successfully used to monitor and modulate activity of specific neuronal and non-neuronal populations that contribute to the disruptions of slow waves (see below). Uncovering the neural circuit mechanisms that underlie the SWA disruptions could lead to the discovery of novel therapeutic strategies.

## Mechanisms Underlying SWA Disruptions in AD

Observations from human studies demonstrated that disrupted SWA contributed to the impairments of memory consolidation in AD patients. However, the mechanisms that underlie SWA disruptions remain largely unknown. Aβ peptides target synapses and disrupt excitatory and inhibitory neurotransmission leading to neural network dysfunction (Selkoe, 2019). This indicates that, SWA anomalies in aMCI and AD patients might be due to the neuronal network dysfunction resulting from neuronal hyper- and hypoactivity. Animal studies using multiphoton microscopy elucidated deficits in inhibitory tone as one possible mechanism for disrupted SWA (Busche et al., 2008; Kastanenka et al., 2017). Deficits in synaptic inhibition led to neuronal hyperactivity (Busche et al., 2008) and caused desynchronized circuit activity within cortical excitatory neurons (Kastanenka et al., 2017). More than 20% of the layer 2/3 cortical neurons exhibited hyperactivity surrounding Aβ plaques. This hyperactivity was reduced when hyperactive neurons were treated with the gamma-aminobutyric acid A (GABA_A_) agonist diazepam (Busche et al., 2008), while slow oscillations were rescued by topical application of GABA directly onto the somatosensory cortex (Kastanenka et al., 2017). In addition to low GABA levels, the expression of GABA_A_ and GABA_B_ receptors was downregulated in APP mice (Kastanenka et al., 2017). Interestingly, application of either GABA_A_ or GABA_B_ inhibitors disrupted slow oscillations in healthy wild-type animals, mimicking slow wave disruptions in APP mice (Kastanenka et al., 2017). As we indicated earlier, GABAergic neurons play a critical role in the onset of cortical silent states, the major element of SWA. Topical applications of a GABA_A_ receptor agonist rescued slow waves and sleep-dependent memory consolidation in transgenic mice (Busche et al., 2015). Thus, APP mice exhibit cortical hyper- and hypoactivity due to deficits in inhibitory elements of the circuit, specifically GABA, GABA_A_, and GABA_B_ receptors, the activity of which is necessary and sufficient for normal SWA.

Alzheimer’s disease is a truly progressive disorder. Deficits in inhibitory elements of the circuit were followed by deficits in excitatory elements (Kastanenka et al., 2019). The protein levels of the cortical excitatory neurotransmitter glutamate were examined in APP mice. Glutamate levels were comparable in APP and wild-type littermates at 7 months of age (Kastanenka et al., 2017). However, by 9 months of age, APP mice started showing deficits in glutamate levels (Kastanenka et al., 2019). These findings indicate that the disturbances of synaptic inhibition followed by a deficiency in synaptic excitation within the neuronal circuits may be related to the disruptions of slow oscillations in AD. Furthermore, administration of the glutamate receptor antagonists alleviated hyperactivity in APP mice (Busche et al., 2008). Taken together, inhibition deficits followed by excitation deficits within the slow wave circuits most likely contributed to the disruption of slow oscillations early in the disease progression and impaired sleep-dependent memory formation.

In addition to inhibitory and excitatory neurons, cortico-thalamic circuits rely on astrocytes to maintain their normal function (Araque et al., 1999; Poskanzer and Yuste, 2011, 2016). Astrocytes are the glial cells that maintain glutamate and GABA recycling via glutamate/GABA-glutamine cycles. Astrocytes form the tripartite synapses with pre- and post-synaptic neuronal compartments to regulate synaptic transmission via astrocytic calcium signaling (Araque et al., 1999; Newman, 2003). Astrocytic contributions to normal circuit function has been underappreciated until recently (Clarke and Barres, 2013; Kastanenka et al., 2020). Amyloid deposits disrupted astrocytic topology (Galea et al., 2015), and astrocytic calcium dynamics were altered in APP mice (Kuchibhotla et al., 2009). Furthermore, elevations in resting calcium concentrations were reported in astrocytes in APP mice (Kuchibhotla et al., 2009). Thus, aberrant astrocytic activity might contribute to the SWA disruptions in AD. Furthermore, the protein expression levels of glutamate transporters GLAST and GLT-1, which localize specifically to astrocytic plasma membrane, were decreased in the cortex and hippocampus in a mouse model of AD (Schallier et al., 2011). Alterations in astrocytic elements of the circuit were also reported in brain tissue from AD patients. The expression of astrocytic glutamine synthetase was decreased in close proximity to Aβ plaques in AD brains (Robinson, 2000). Interestingly, aberrant expression of glutamine synthetase was detected in a subpopulation of pyramidal neurons in AD individuals (Robinson, 2000), suggesting that the glutamate-glutamine cycle was disrupted. The abnormalities in astrocytic activity may contribute to aberrant neuronal firing and lead to the disruption of neuronal networks, thus perturbing SWA. Indeed, astrocytes participated in triggering slow oscillation UP states *in vitro* (Poskanzer and Yuste, 2011) and *in vivo* (Poskanzer and Yuste, 2016; Szabó et al., 2017). Poskanzer and Yuste (2016) visualized intracellular calcium transients and demonstrated that astrocytes had modulated extracellular glutamate, thus triggering the SWA in mouse brains. Furthermore, Szabó et al. (2017) showed that blocking astrocytic calcium transients resulted in reduced numbers of astrocytes and neurons participating in the SWA. This series of studies supports the idea that astrocytes are necessary and play a critical role in induction of slow oscillation UP states in the cortical circuits. Therefore, astrocytic network studies using animal models are important to understand the role of astrocytes in slow wave dysfunction in AD. Finally, understanding the role of astrocytes in SWA disruptions might point to a novel therapeutic strategy for Alzheimer’s patients.

## Optogenetic Control of Slow Oscillations in Mouse Models of AD

Optogenetics is a leading-edge research tool that can be used to gain valuable insight into the causal relationship between circuit dynamics and Alzheimer’s progression using animal models (Boyden et al., 2005; Li et al., 2005). This methodology provides high spatiotemporal precision with cell-type specificity. Distinct cell types can be targeted *in vivo* using cell-type specific promoters with light-activatable channels or proton pumps (Fenno et al., 2011). Light activation of cells expressing the channels or pumps can be used to manipulate the activity within neural circuits of interest. Optogenetics has been successfully adopted to studies of AD using mouse models.

Optogenetics was used to increase neuronal activity chronically in hippocampal perforant pathway in AD mice. This exacerbated hyperactivity in the circuit and increased interstitial fluid Aβ42 levels as well as Aβ deposition in the projection areas (Yamamoto et al., 2015). Optogenetic-mediated increases in neuronal activity also elevated release and propagation of tau in htau mice (Wu et al., 2016). This evidence further solidified the fact that aberrant synaptic activity facilitated AD progression (Cirrito et al., 2005, 2008). Optogenetics was also used to shed light onto the state of brain rhythms in AD. Optogenetic entrainment of interneurons in the gamma frequency range restored gamma oscillations and reduced Aβ deposition in a mouse model of AD (Iaccarino et al., 2016). Our laboratory reported that light-activation of channelrhodopsin-2 (ChR2)-expressing excitatory neurons at the endogenous frequency of slow waves in APP mice for 4 weeks rescued aberrant slow oscillations by restoring slow wave power. It also restored GABA as well as GABA_A_ and GABA_B_ receptor levels (Kastanenka et al., 2017). In addition, chronic restoration of SWA halted Aβ plaque deposition and prevented intraneuronal calcium elevations (defined as calcium overload) (Kastanenka et al., 2017). Alternatively, driving slow waves at twice the endogenous frequency using optogenetics augmented Aβ production, increased neuronal calcium overload and decreased the synaptic spine density (Kastanenka et al., 2019). Optogenetic restoration of circuit activity slowed pathology progression in mouse models of AD, while optogenetic increases in the frequency of slow waves accelerated the progressive pathophysiology and resulted in neuronal network failure.

Similarly, modulations of brain wave activity restored memory deficits in experimental models using optogenetics. Synchronization of SWA in somatosensory and motor cortices using optogenetics was able to restore perceptual memory impairment and prolong memory retention in sleep-deprived mice (Miyamoto et al., 2016). In addition, restoration of hippocampal oscillations with optogenetics resulted in an improvement of recognition memory in APP mice (Giovannetti et al., 2018). Also, optogenetic activation of memory engram cells in hippocampus increased spine density in engram cells and restored long-term memory (Roy et al., 2016). Furthermore, gamma oscillation rescue using optogenetics improved spatial memory in an AD mouse model (Etter et al., 2019). Restoring brain oscillations by optogenetic approaches in mouse models provides insight into novel therapeutic approaches to treat and/or prevent AD altogether. Animal studies suggest that restoring brain oscillation activity, including SWA, may be an effective therapeutic strategy for reducing memory deficits in AD patients.

## Restoring Slow Wave Sleep Is a Promising Therapy for AD

Currently, there are no effective treatments able to slow AD progression and alleviate cognitive and memory impairments in patients. The majority of clinical therapeutic approaches focus on clearing Aβ and tau with monoclonal antibodies using passive immunotherapies (van Dyck, 2018). Light therapy had mixed results in the clinic (Dowling et al., 2005, 2008; Riemersma-van der Lek et al., 2008). However, a large number of clinical trial failures underscores the need to identify novel therapeutic strategies for treating AD.

Restoration of SWA during NREM sleep in Alzheimer’s patients might slow the disease progression and rescue sleep-dependent memory consolidation. Transcranial direct current stimulation (tDCS) and transcranial magnetic stimulation (TMS) are two noninvasive brain stimulation methodologies that could potentially be used to do so. Recently, tDCS was applied to human MCI subjects during daytime nap to investigate the patterns of SWA and sleep-dependent memory consolidation. Both the slow oscillation power and memory performance were improved after stimulating the brain at the slow oscillation frequency with tDCS (Ladenbauer et al., 2017). Furthermore, repeated applications of tDCS induced slow oscillations during SWS and led to enhanced declarative memory retention the next day in older (Westerberg et al., 2015) and in young healthy adults (Marshall et al., 2004, 2006). In a similar study, TMS was used to evoke slow waves during NREM sleep. TMS increased SWA power in healthy young subjects (Massimini et al., 2007). In addition to tDCS and TMS methodologies, slow oscillations can be enhanced with auditory stimulation (Ngo et al., 2013; Leminen et al., 2017; Papalambros et al., 2017; Ong et al., 2018). Applied auditory tones that were phase-locked to the up states of slow oscillations during sleep benefited declarative memory consolidation in healthy young adults (Ngo et al., 2013; Leminen et al., 2017) and in older subjects (Papalambros et al., 2017). Furthermore, phase-locked acoustic stimulation also enhanced memory encoding during nap in healthy young subjects (Ong et al., 2018). Another sensory stimulation strategy visual stimulation, can be used to induce SWA (Riedner et al., 2011). Using high-density EEG recordings in healthy young subjects during NREM sleep, SWA was successfully evoked by visual stimuli (Riedner et al., 2011). Thus, tDCS, TMS, acoustic, and visual stimulations could potentially be used to enhance sleep-dependent memory consolidation in healthy subjects and AD patients in early stages of the disease.

Targeting specific GABAergic neuronal circuit elements may be particularly attractive in designing new AD therapies. Whereas restoration of parvalbumin interneuron activity acutely restored gamma oscillations (Iaccarino et al., 2016) and prevented memory loss as well as network hyperexcitability (Hijazi et al., 2019), activation of either neuronal nitric oxide synthase (nNOS) or somatostatin neurons may be useful for restoring SWA. A recent study involving the chemogenetic activation of SST-positive cells in the cerebral cortex showed increased SWA, elevated slope of individual slow waves, and prolonged NREM sleep duration compared to control conditions. Alternatively, chemogenetic inhibition of these cells reduced SWA and slow-wave incidence without changing time spent in NREM sleep (Funk et al., 2017). We previously demonstrated that nNOS neurons are activated during episodes of NREM sleep associated with increased SWA (Gerashchenko et al., 2008). We also showed that optogenetically evoked responses in nNOS-positive cells of the cerebral cortex are consistent with their role in slow-wave sleep physiology (Gerashchenko et al., 2018). Furthermore, mice lacking nNOS expression in SST positive neurons exhibited significant impairments in both homeostatic low delta frequency range SWA production and a recognition memory task that relies on cortical input (Zielinski et al., 2019). Further studies will determine whether activation of nNOS/somatostatin neurons in the cerebral cortex is efficient in reducing AD pathology, and whether this effect is mediated by SWA enhancement. Finally, instead of activating endogenous interneurons, it would be promising to explore cell-based therapeutic strategies, such as transplantation of human stem cell-derived interneurons to increase inhibitory tone and restore SWA.

## Discussion

In addition to memory disruptions, Alzheimer’s patients experience disturbances in their sleep-wake cycles, due to increased nighttime wakefulness and decreased NREM SWS. AD pathology is correlated with SWA disruptions at the early stages of AD. Decreased SWA was found in asymptomatic cognitively normal adults and aMCI patients. Since slow oscillation disruption is an early event, it has the potential to be used as an early biomarker for AD. It should be noted that a lot of human studies discussed here were based on a low sample size at higher risk for false positives due to random variations in a small number of data points. Thus replications are needed to validate the findings. Nevertheless, disruptions in slow oscillations might underlie the memory impairments as part of AD progression, since SWA plays a key role in declarative memory consolidation during sleep. Moreover, animal models of AD recapitulate the slow wave disruptions and can be used for mechanistic studies. Use of leading-edge technologies, including optogenetics, wide-field imaging and multiphoton microscopy, in addition to traditional technologies, including electrophysiology, provided insight into the mechanisms of slow wave disruptions in AD. A better understanding of the relationship between SWA disruptions and memory decline may shed light on the mechanistic pathways underlying AD-associated memory impairment. SWA restoration provides a promising novel therapeutic target for AD. Utilizing noninvasive brain stimulation technologies and medications that upregulate inhibitory elements of cortico-thalamic circuits may prove to become efficient therapeutic strategies. Development of novel therapeutic interventions targeting SWA during NREM sleep early in the disease progression might slow memory decline in the elderly and delay AD onset in MCI or healthy individuals at risk for developing AD.

## Author Contributions

YL and KK wrote the original draft of the manuscript. YL and KK prepared the figure. YL, DG, IT, BB, and KK reviewed and edited the final manuscript. All authors contributed to the article and approved the submitted version.

## Conflict of Interest

The authors declare that the research was conducted in the absence of any commercial or financial relationships that could be construed as a potential conflict of interest.
